# ITS1-based profiling of the skin mycobiome in truncal acne reveals altered baseline ecology and heterogeneous doxycycline-associated patterns

**DOI:** 10.71150/jm.2512013

**Published:** 2026-02-28

**Authors:** Nayan Jin, Woo Jun Sul, Hye Rim Do, Hei Sung Kim

**Affiliations:** 1 Department of Dermatology, Incheon St. Mary's Hospital, The Catholic University of Korea, Seoul 06591, Republic of Korea; 2 Systems Microbial Ecology Laboratory, Department of Systems Biotechnology, Chung-Ang University, Anseong 17546, Republic of Korea

**Keywords:** ITS1, truncal acne, mycobiome

## Abstract

Truncal acne represents a biologically distinct manifestation of acne vulgaris, yet its fungal ecology remains incompletely characterized. Previous work using internal transcribed spacer 2 (ITS2) sequencing suggested that truncal acne is associated with altered fungal richness and *Malassezia* species composition; however, fungal marker choice may influence ecological inference, particularly in sebaceous skin dominated by *Malassezia*. In this study, we characterized the truncal skin mycobiome of patients with truncal acne and healthy controls using internal transcribed spacer 1 (ITS1) amplicon sequencing. Skin swabs were collected from the upper back, and fungal communities were analyzed using QIIME 2 with taxonomic assignment against the UNITE *v10.0* database. Baseline acne–control differences and doxycycline-associated patterns were evaluated using alpha- and beta-diversity metrics and differential abundance analyses. Doxycycline-associated patterns were assessed using paired, within-patient pre- and post-exposure comparisons. ITS1 profiling demonstrated that truncal acne was associated with altered baseline fungal ecology compared with controls, characterized by reduced alpha diversity and ASV-level differences within *Malassezia*-dominated communities. Beta-diversity analyses showed substantial overlap between acne and control samples, indicating limited global separation. Following doxycycline exposure, fungal communities remained *Malassezia*-dominant and did not demonstrate uniform convergence toward control profiles; instead, species- and ASV-level differences were heterogeneous across individuals and exposure durations. Together with prior ITS2-based findings, these results underscore the importance of marker-dependent perspectives when interpreting fungal ecology in sebaceous skin.

## Introduction

Truncal skin is among the most sebaceous and environmentally constrained regions of the human body, characterized by large follicular units, limited ventilation, and frequent occlusion from clothing. In individuals with acne vulgaris, these features create a cutaneous environment that differs from facial skin and may support distinct microbial assemblages (Aly et al., 1978; Otberg et al., 2004; Sandby-Møller et al., 2003). Despite the clinical burden of truncal acne (Ballanger et al., 2023; Menteşoğlu et al., 2025), microbial studies have historically prioritized facial sites, leaving truncal microbial ecology comparatively underexplored.

Fungal communities represent a major but incompletely understood component of the truncal skin ecosystem (Grice and Segre, 2011; Prohic et al., 2016). In sebaceous regions, fungal biomass is dominated by lipid-dependent members of the genus *Malassezia*, which are adapted to utilize host-derived lipids and persist under conditions of limited nutrient diversity. *Malassezia* species differ substantially in enzymatic repertoires, lipid-processing capacity, and host immune interactions (Li et al., 2022), suggesting that subtle shifts in community structure—rather than overt overgrowth—may influence inflammatory states (Ayers et al., 2005; Findley et al., 2013; Martínez-Ortega et al., 2024; Park et al., 2021a, 2021b). However, whether truncal acne is associated with alterations in the dominant fungal core versus expansion of rare taxa remains unclear.

Systemic antibiotics, particularly doxycycline, are a cornerstone of therapy for moderate-to-severe truncal acne and are prescribed primarily for antibacterial and anti-inflammatory effects (Woo and Kim, 2022). Although doxycycline has no clinically meaningful antifungal activity, antibiotic exposure may indirectly influence fungal communities by altering bacterial–fungal interactions, sebum availability, or local immune tone. Whether such perturbations reshape the truncal mycobiome, or whether dominant fungal communities remain resilient under antibiotic pressure, has not been well characterized.

Amplicon-based profiling of fungal communities is inherently influenced by marker selection, especially in low-diversity, *Malassezia*-dominated niches. Different internal transcribed spacer (ITS) regions vary in amplification bias and taxonomic resolution and may emphasize different ecological features of the same community. We previously examined the truncal acne mycobiome using ITS2 sequencing and observed altered fungal richness and species composition in acne lesions. To further interrogate community structure and stability in this setting, we extended this work using ITS1 sequencing, which may preferentially capture dominant and persistent fungal residents.

Here, we used ITS1 amplicon sequencing to characterize baseline truncal mycobiome ecology in acne patients compared with controls and to examine doxycycline-associated patterns using paired, within-patient comparisons. By focusing on dominant community structure and antibiotic-associated deviations from baseline, this study provides a complementary ecological perspective on truncal acne and highlights how marker choice influences interpretation of fungal dynamics in sebaceous skin.

## Materials and Methods

### Study population and antibiotic exposure classification

Patients with truncal acne and healthy controls were recruited from Incheon St. Mary’s Hospital, The Catholic University of Korea. Moderate-to-severe truncal acne (Investigator’s Global Assessment [IGA] grades 3–4) involving the chest, shoulders, and/or back was diagnosed by board-certified dermatologists. Healthy controls had no history of truncal acne or other inflammatory skin diseases.

Participants were excluded if they had received systemic or topical antibiotics or antifungals within the preceding three months, as antimicrobial exposure can induce sustained alterations in cutaneous microbial and fungal community structure. Individuals with prior isotretinoin therapy were excluded because of its long-lasting effects on sebaceous gland activity and follicular microenvironments that influence microbial colonization. Pregnant individuals were excluded due to hormonal and immunologic changes affecting skin physiology. Finally, individuals with other inflammatory skin diseases or chronic inflammatory or immunologic conditions were excluded to minimize confounding effects on cutaneous microbiome and mycobiome profiles. Truncal acne was clinically distinguished from *Malassezia* folliculitis based on lesion morphology and distribution, including the absence of monomorphic pruritic papules or pustules, and an overall presentation inconsistent with fungal folliculitis.

The study was approved by the institutional review board (OC21TISI0158), and all participants provided written informed consent.

Skin swab samples were collected from the upper back using a standardized protocol. All 17 acne patients provided paired baseline (V0) and post-doxycycline samples, with follow-up obtained at variable timepoints (10 patients at V1, 7 at V2, and 1 at V3; one individual contributed samples at more than one follow-up visit) (Table S1). Follow-up visits corresponded to oral doxycycline 100 mg administered twice daily for approximately 6 weeks (V1), 12 weeks (V2), and 18 weeks (V3). Because follow-up timing varied among patients, doxycycline-associated analyses were interpreted as paired, within-subject exposure comparisons rather than formal longitudinal trajectories.

### Genomic DNA extraction from skin swabs

Fungal genomic DNA was extracted from skin swab samples using the PureLink^®^ Genomic DNA Mini Kit (Invitrogen, Thermo Fisher Scientific) with bead-beating–assisted lysis. Swabs were incubated in lysozyme digestion buffer at 37°C for 1 h, mechanically disrupted with a stainless-steel bead and proteinase K, and incubated at 55°C for 30 min. DNA purification was performed according to the manufacturer’s protocol, with gDNA eluted in 30 µl elution buffer. DNA quantity and purity were assessed using NanoDrop 2000 and Qubit 4 fluorometers.

### Mycobiome amplicon sequencing and library preparation

The ITS1 region was amplified using the 18SF and 5.8S 1R primers containing Illumina adapter overhangs. PCR reactions (25 µl) were performed using KAPA HiFi HotStart ReadyMix with standardized cycling conditions. Amplicons were purified with AMPure XP beads, indexed using the Nextera XT Index Kit (Illumina), pooled, and sequenced on an Illumina MiSeq platform using paired-end 2 × 300 bp chemistry.

### Bioinformatics analysis

Sequence data were processed using QIIME 2 (version 2024.10). Paired-end reads were imported, primer sequences were removed using cutadapt, and denoising, dereplication, and merging were performed with DADA2. From 1,114,203 raw reads, 936,628 merged reads were retained to generate an amplicon sequence variant (ASV) table at 100% sequence identity. A phylogenetic tree was constructed using the align-to-tree-mafft-fasttree pipeline, and taxonomy was assigned using a pretrained classify-sklearn classifier against the UNITE database (version 10.0, February 2025 release).

### Statistical analysis

Statistical analyses were conducted using QIIME 2 (version 2024.10) and R (version 4.3.2) with the vegan package (version 2.6-10). The ASV table was rarefied to 18,000 reads per sample prior to diversity analyses. Alpha diversity (observed features, Shannon entropy, Pielou’s evenness, and Faith’s phylogenetic diversity) was compared between groups using the Wilcoxon rank-sum test. Beta diversity was assessed using Bray–Curtis dissimilarity and unweighted and weighted UniFrac distances, visualized by principal coordinates analysis, and tested using PERMANOVA.

Because ITS1 is a non-coding, highly variable region with frequent indels, ITS-derived multiple sequence alignments and inferred branch lengths are inherently approximate and should not be interpreted as definitive evolutionary phylogenies. Accordingly, Faith’s PD and UniFrac are reported as tree-informed proxy metrics (sensitivity analyses) to complement non-phylogenetic measures (e.g., Bray–Curtis), and interpretations emphasize concordance across metrics rather than phylogenetic inference from ITS-derived trees.

For doxycycline-associated analyses, pre- and post-exposure samples were compared within matched patients. Because the study was not designed or powered for formal longitudinal modeling, these analyses were interpreted as paired, within-subject exposure comparisons. Differentially abundant ASVs were identified using LEfSe (Kruskal–Wallis test, *p* < 0.05). An LDA threshold of 3.0 was applied for baseline acne–control comparisons, while lower thresholds (LDA > 2.0) were used for doxycycline-associated analyses, which were interpreted as exploratory and hypothesis-generating. Because LEfSe screens a large number of features and we did not apply a formal multiple-testing correction, ASV-level LEfSe results are presented as exploratory/hypothesis-generating candidates rather than definitive biomarkers. A two-sided *p*-value < 0.05 was considered statistically significant.

## Results

### Baseline truncal acne mycobiome differs from healthy controls

**Fig. 1.**
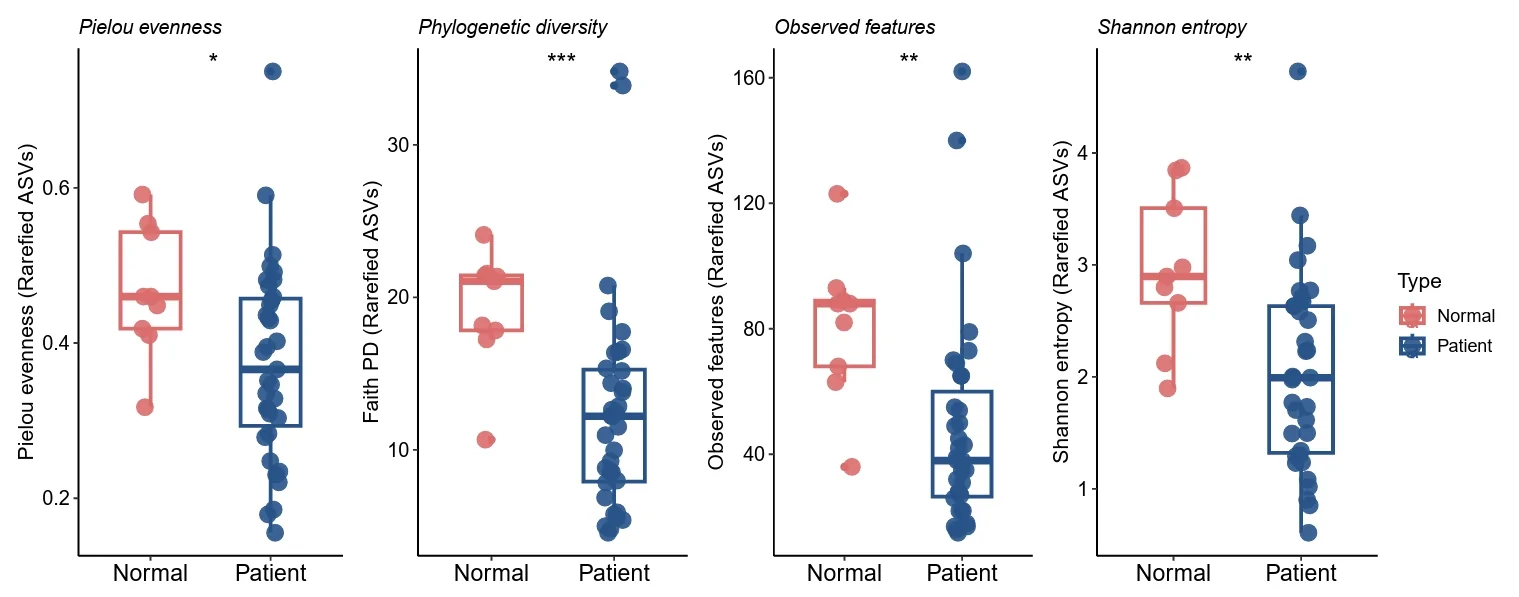
Baseline truncal acne mycobiome differs from healthy controls. ITS1-based alpha-diversity analysis demonstrated significantly lower fungal diversity in acne samples compared with controls, as reflected by reduced observed ASVs, Shannon diversity, Pielou’s evenness, and Faith’s phylogenetic diversity.

At baseline (V0), truncal skin fungal communities in both acne patients and healthy controls were dominated by members of the genus *Malassezia*. ITS1-based alpha-diversity analysis demonstrated significantly lower fungal diversity in acne samples compared with controls, as reflected by reduced observed ASVs, Shannon diversity, Pielou’s evenness, and Faith’s phylogenetic diversity (Fig. 1). These findings indicate that truncal acne is associated with altered baseline fungal ecology characterized by reduced richness and evenness within *Malassezia*-dominated communities.

### Beta diversity reveals relative abundance-driven differences with shared dominant taxa

**Fig. 2.**
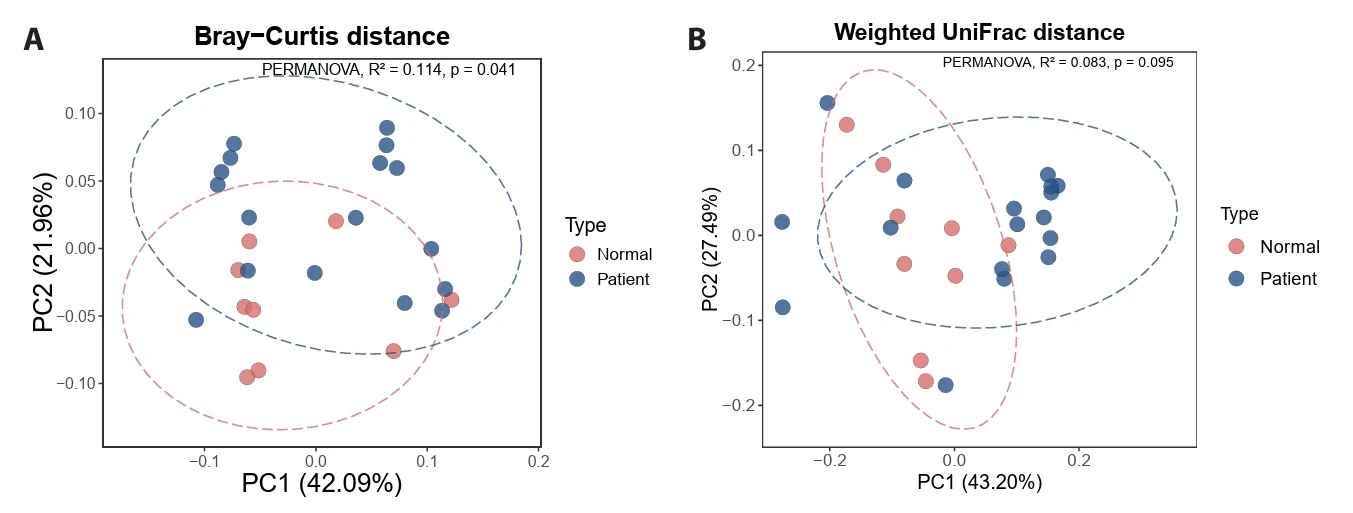
Beta diversity reveals relative abundance-driven differences with shared dominant taxa.

Principal coordinates analysis based on Bray–Curtis dissimilarity revealed a statistically significant difference in fungal community composition between baseline acne and control samples (PERMANOVA, *p* < 0.05) (Fig. 2A). In contrast, UniFrac-based analyses demonstrated substantial overlap between groups, with weaker separation observed for weighted UniFrac distances (Fig. 2B). Given the approximate nature of ITS-derived trees, UniFrac results are interpreted as complementary, tree-informed sensitivity analyses rather than definitive evidence of phylogenetic restructuring. This pattern indicates that truncal acne is associated with differences in relative abundance and ASV composition rather than replacement of dominant fungal taxa, consistent with perturbation of a shared fungal core.

### Taxonomic composition and differential ASV abundance at baseline

**Fig. 3.**
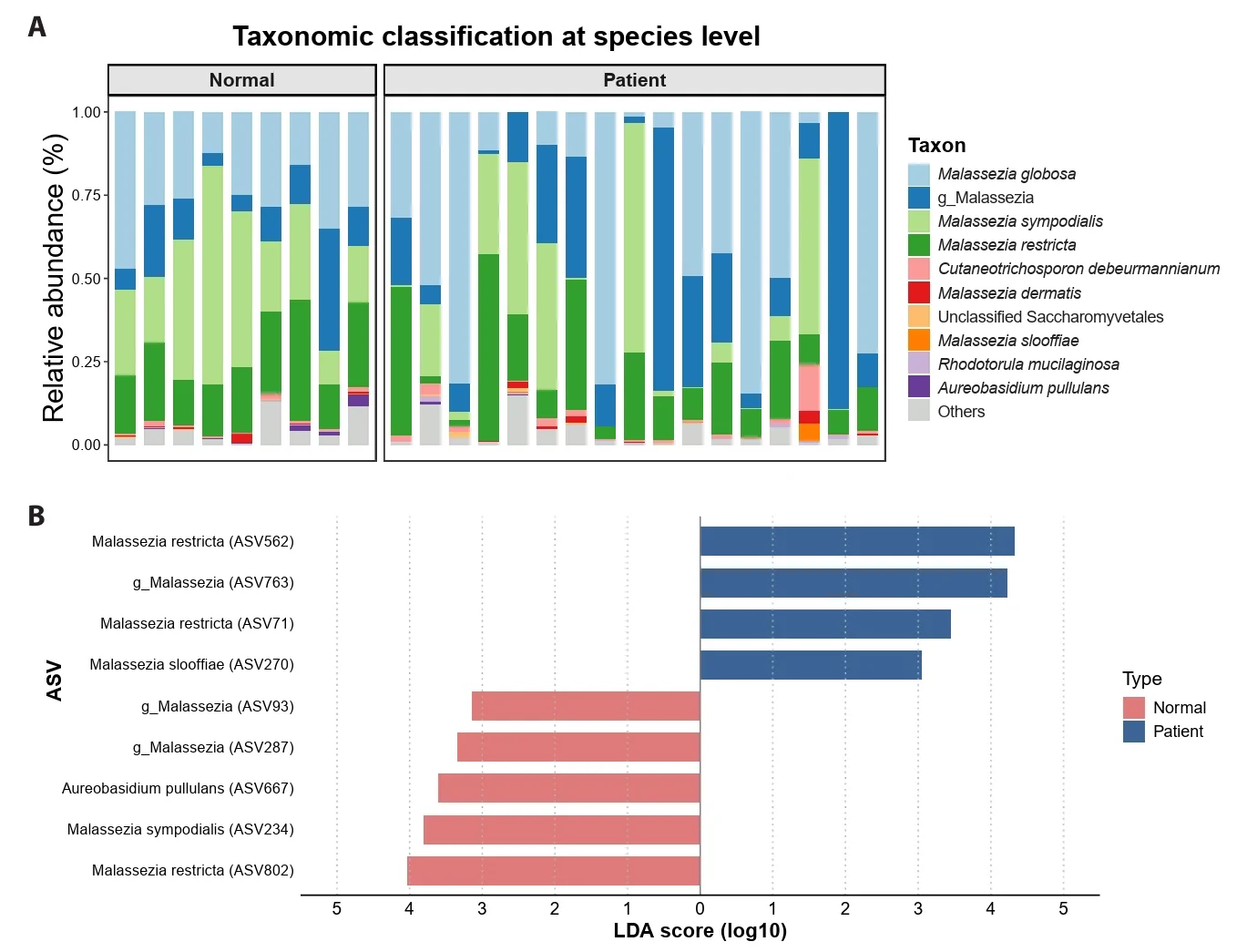
Taxonomic composition and differential ASV abundance at baseline.

Species-level taxonomic profiling confirmed strong *Malassezia* dominance in both acne and control samples (Fig. 3A). Although overall genus-level composition was similar, acne samples exhibited greater heterogeneity in species and ASV distribution. LEfSe analysis identified multiple candidate ASVs that were differentially abundant between acne and control groups (Kruskal–Wallis *p* < 0.05, LDA > 3.0) (Fig. 3B).

### Doxycycline exposure is not associated with global restructuring of the truncal mycobiome

**Fig. 4.**
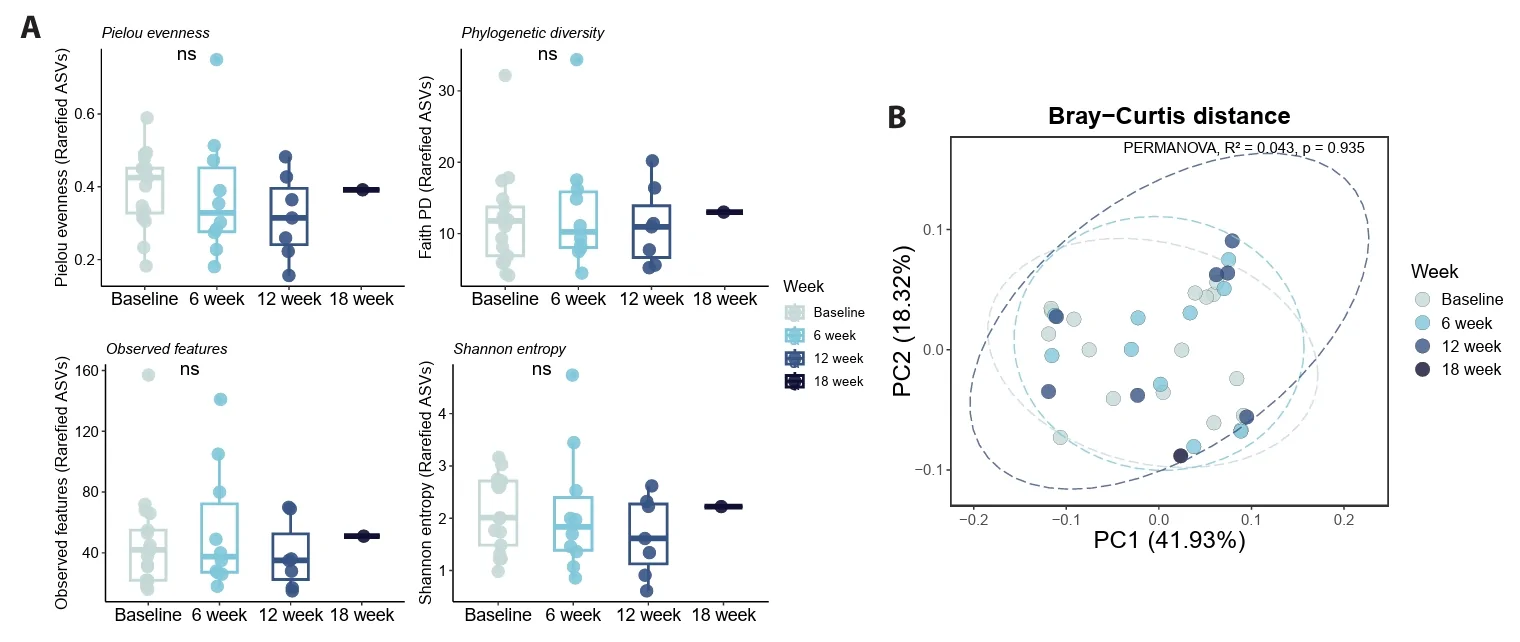
Global truncal mycobiome structure is stable after doxycycline exposure.

All acne patients provided paired baseline (V0) and post-treatment samples following oral doxycycline therapy (100 mg twice daily), with follow-up categorized as V1 (6 weeks), V2 (12 weeks), or V3 (18 weeks). Across exposure durations, no significant changes in fungal alpha diversity were observed in paired pre- and post-treatment comparisons (Fig. 4A), indicating stability of overall fungal richness and evenness following antibiotic exposure.

Beta-diversity analyses further supported community stability following doxycycline treatment. Ordination based on Bray–Curtis and UniFrac distances showed substantial overlap between pre- and post-treatment samples, with no statistically significant global separation detected (PERMANOVA and ANOSIM, *p* > 0.05) (Fig. 4B). These findings suggest that doxycycline exposure does not induce large-scale restructuring of the truncal fungal community.

### Doxycycline exposure is associated with heterogeneous ASV-level differences

**Fig. 5.**
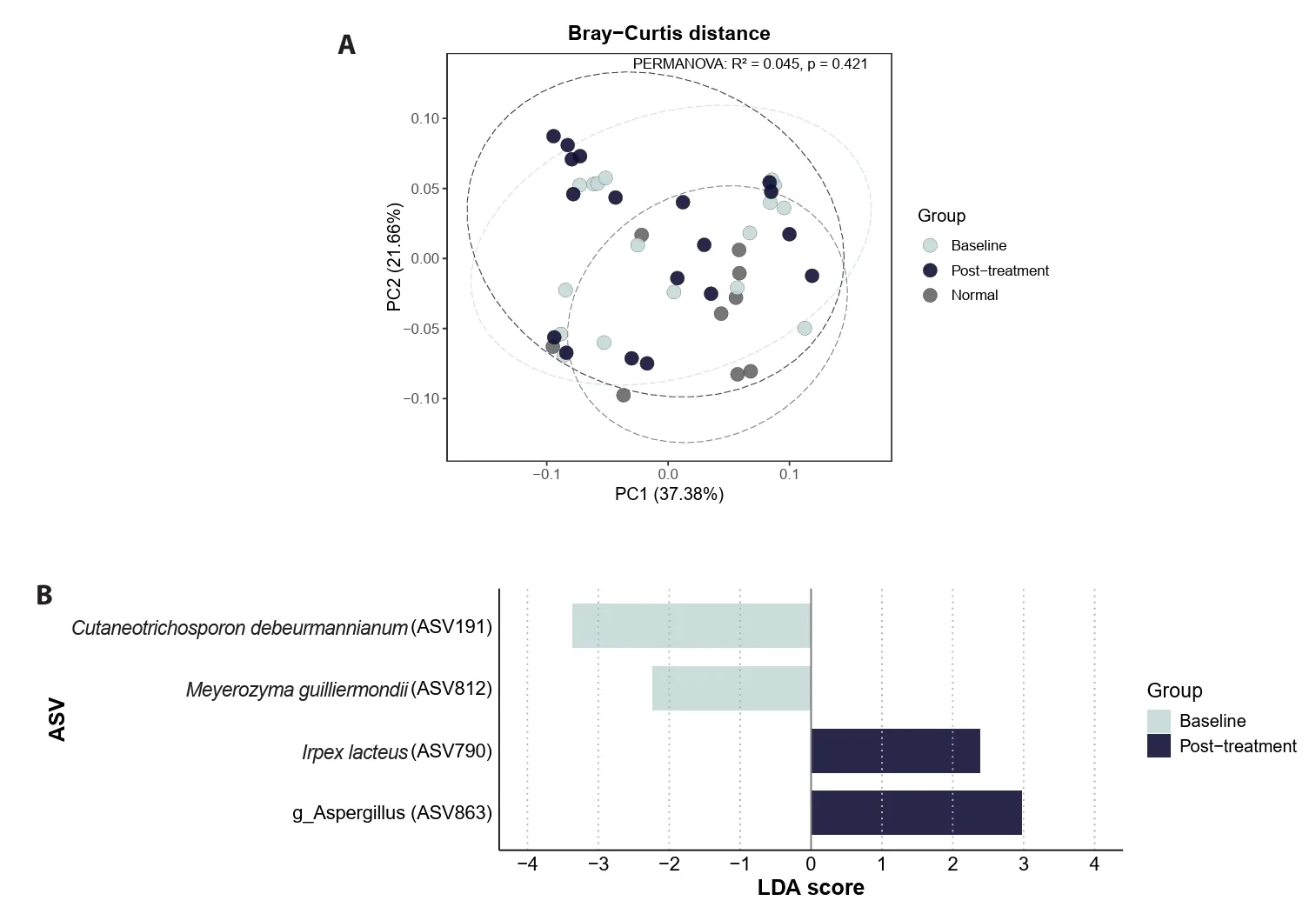
Heterogeneous ASV-level differences after doxycycline exposure.

Paired comparisons between baseline (V0) and post-doxycycline samples (V1, V2, or V3) revealed heterogeneous species- and ASV-level differences following antibiotic exposure. However, these post-treatment communities did not demonstrate uniform convergence toward control-like profiles and were not accompanied by consistent shifts in global beta-diversity structure (Fig. 5A). LEfSe analysis using a lower threshold (Kruskal–Wallis *p* < 0.05, LDA > 2.0) identified exploratory, hypothesis-generating ASV-level candidates associated with doxycycline exposure status (Fig. 5B).

## Discussion

Using ITS1 amplicon sequencing, we demonstrate that truncal acne is associated with altered baseline fungal ecology compared with healthy truncal skin, characterized by reduced alpha diversity and ASV-level differences within *Malassezia*-dominated communities. These findings suggest perturbation within a largely conserved fungal framework rather than replacement of core taxa, indicating that disease-associated differences occur primarily at finer taxonomic resolution.

Doxycycline-associated analyses in this study reflect paired, within-patient comparisons between baseline (V0) and post-treatment samples obtained after clinically relevant exposure durations. Across exposure groups, fungal communities remained *Malassezia*-dominant and did not exhibit global restructuring or convergence toward control profiles, indicating relative resilience of the truncal mycobiome to indirect antibiotic pressure. Nevertheless, paired analyses identified heterogeneous ASV-level differences across individuals, consistent with fine-scale variation rather than uniform community remodeling. Accordingly, doxycycline-associated findings should be interpreted cautiously.

An important consideration in interpreting these results is the choice of fungal marker. The ITS1-based patterns observed here differ in several respects from our prior ITS2-based analysis of the same cohort, which identified increased fungal richness and clearer beta-diversity separation in truncal acne. Rather than representing contradictory findings, these differences likely reflect marker-dependent resolution of distinct ecological layers within *Malassezia*-dominated communities. In sebaceous skin, where fungal diversity is inherently low and communities are dominated by lipid-dependent taxa, ITS1 profiling may preferentially capture dominant and stable fungal residents, providing a conservative representation of core community structure. In contrast, ITS2-based approaches may be more sensitive to low-abundance or transient taxa and thus accentuate differences in richness or community separation. Viewed together, ITS1 and ITS2 offer complementary, marker-dependent perspectives on truncal fungal ecology rather than interchangeable measures (Bellemain et al., 2010; Findley et al., 2013; Jagielski et al., 2014; Jo et al., 2016).

This study has several limitations, including variable follow-up timing, absence of bacterial or functional profiling, and lack of direct sebum measurements. In addition, potential truncal-specific confounders—such as bathing habits, topical product use, clothing occlusion, physical activity, and seasonality—were not formally controlled. Finally, the control cohort was female-predominant, which may limit inference regarding sex-associated variation. Future studies incorporating longitudinal designs, multi-kingdom profiling, and sex-balanced control cohorts are warranted to further clarify fungal dynamics in truncal acne. In addition, phylogeny-informed metrics derived from ITS1 amplicons rely on approximate alignments and inferred trees; therefore, Faith’s PD and UniFrac are interpreted as complementary, tree-informed proxies rather than definitive phylogenetic estimates.

## Supplementary Information

Table S1. Demographic data on the truncal acne and control population
