# Supplementary material for: ITS1-based profiling of the skin mycobiome in truncal acne reveals altered baseline ecology and heterogeneous doxycycline-associated patterns
Source: J Microbiol. 2026 Feb 28;64(2):e2512013. doi: 10.71150/jm.2512013 (PMC13577111; doi:10.71150/jm.2512013)
Supplement: Supplementary file 1 [file jm-2512013-Supplementary-Table-S1.pdf]

Table S1. Demographic data on the truncal acne and control population

Truncal acne patients

No.	Sample No.	Sex	Age	V0	V1	V2	V3
1	4	M	20	O		O	
2	6	M	17	O		O	
3	7	M	22	O	O		
4	8	F	13	O		O	
5	11	M	21	O	O		
6	12	M	29	O	O		
7	15	M	25	O		O	
8	16	M	45	O	O		
9	17	F	48	O		O	
10	18	F	16	O			O
11	19	F	18	O		O	
12	20	M	17	O	O		
13	21	M	53	O	O		
14	22	M	34	O	O	O	
15	23	F	30	O	O		
16	24	M	27	O	O		
17	25	M	43	O	O		

Control population

No.	Sample No.	Sex	Age
1	1	M	31
2	2	F	31
3	3	F	27
4	4	F	29
5	5	F	48
6	6	F	49
7	7	F	28
8	9	F	35
9	10	F	45
